# Long COVID-19 Syndrome: A Comprehensive Review of Its Effect on Various Organ Systems and Recommendation on Rehabilitation Plans

**DOI:** 10.3390/biomedicines9080966

**Published:** 2021-08-05

**Authors:** Zhipeng Yan, Ming Yang, Ching-Lung Lai

**Affiliations:** 1 Department of Medicine, Queen Mary Hospital, The University of Hong Kong, Hong Kong 999077, China; 2 Department of Ophthalmology, Li Ka Shing Faculty of Medicine, The University of Hong Kong, Hong Kong 999077, China; hrmeym@hku.hk

**Keywords:** Long COVID-19 Syndrome, Post COVID-19 Syndrome, rehabilitations, recovery

## Abstract

The majority of people infected with SARS-CoV-2 fully recovered within a few weeks. However, a considerable number of patients of different ages still suffer from long-lasting problems similar to the multi-organ damage in its acute phase of infection, or experience symptoms continuously for a longer term after the recovery. The severity of the primary infection seems not to be associated with the possibility and severity of long-term symptoms. Various unresolved symptoms have been reported in COVID-19 survivors months after hospital discharge. Long COVID-19 Syndrome refers to survivors 4 months after initial symptoms onset. It is important to understand the systemic effects of Long COVID-19 Syndrome, its presentations, and the need for rehabilitations to restore functional recovery in survivors. Government, healthcare workers, and survivor groups should collaborate to establish a self-sustaining system to facilitate follow-up and rehabilitations, with prioritization of resources to more severely Long COVID-19 Syndrome survivors. This review looks into the systemic effects of Long COVID-19 Syndrome in various aspects: respiratory, cardiovascular, hematological, renal, gastrointestinal, neurological, and metabolic effects of Long COVID-19 Syndromes. Recommendations for follow-up and rehabilitations details have been explored to cope with the tremendous Long COVID-19 Syndrome patients.

## 1. Introduction

Ever since the first case of COVID-19 reported in early December 2019 in China, the incidence has been rising drastically. As of 11 May 2021, the total number of recorded infections is over 157 million, with over 3.2 million deaths [1,2]. Though a majority of patients recovered from COVID-19 infections, over 70%-of-survivors were reported to have impairments in one or more organs 4 months after initial symptoms [3]. They are termed “long haulers” [4], or patients living with “Chronic COVID syndrome”, “post-COVID-19 syndrome”, or “postacute-COVID19 [5,6]. Extensive symptoms have been reported by convalescent patients, such as chronic cough, chest tightness, shortness of breath, cognitive dysfunction, and extreme fatigue, in Long COVID-19 Syndrome [7].

**Figure 1:**
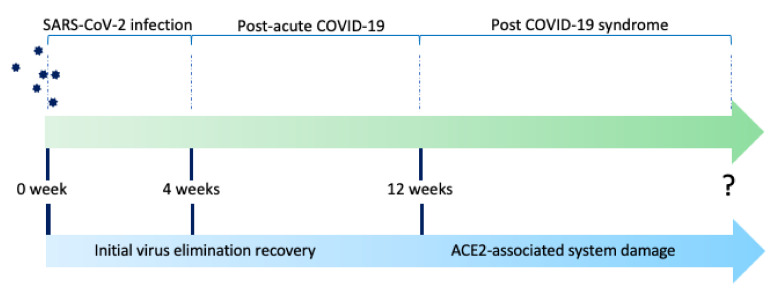
The timeline of Post-acute COVID-19 syndrome is defined as 4 to 12 weeks between initial confirmation of SARS-CoV-2 infection, while Post-COVID-19 syndrome is defined as 12 weeks after initial infection. The underlying progression/persistence of symptoms is associated with initial virus elimination recovery and angiotensin-converting enzyme 2 (ACE2)-associated system damage.

Long COVID-19 Syndrome is an umbrella term, including post-acute COVID-19 and post-COVID-19 syndrome, depending on the duration after the acute onset of symptoms (Figure 1). Postacute-COVID-19 is defined as ongoing symptomatic COVID-19 for people who still have symptoms 4 and 12 weeks after the onset of acute symptoms, while post-COVID-19-syndrome is for people who still have symptoms for more than 12 weeks after onset of acute symptoms according to the UK NICE guidelines [7]. In a systematic review and meta-analysis looking into the long-term effect of COVID-19, the five most common symptoms are fatigue (58%), headache (44%), attention disorder (27%), hair loss (25%), and dyspnea (24%) [8]. These symptoms may take months to resolve, even among non-hospitalized persons with mild illness course in the acute phase [9].

Despite the commencement of global COVID-19 vaccination programs [10], the infection rate remains high at the time of writing. This may imply we will have to live with COVID-19 for a significant period of time before there is herd immunity. It is important to understand the long-term effects of COVID-19 infection and manage its possible long-term complications in recovered COVID-19 patients to facilitate follow-up treatments.

COVID-19 is caused by SARS-CoV-2 infection. SARS-CoV-2 uses the angiotensin-converting-enzyme 2 (ACE2) receptor to enter the cell through binding with spike-like protein (S-protein) [11,12], though some other receptors may also be involved. ACE2, therefore, plays a vital role in the pathogenesis of COVID-19. ACE2 is widely expressed in different body tissues, including the lung, heart, liver, kidney, and gastrointestinal system [13]. Thus, multi-organ injuries are observed in COVID-19, such as acute respiratory distress syndrome, acute myocardial injury, acute kidney injury, and acute liver injury. Survivors of severe COVID-19 are also found to be with multi-organ impairments after discharge [5].

Recovered COVID-19 patients are defined as previously infected patients with SARS-COV-2 negative testing by polymerase-chain-reaction (PCR) on 2 consecutive occasions separated by at least 24 h and without clinical symptoms at the time of discharge [14].

## 2. The Effects of Long COVID-19 Syndrome on Various Systems

**Figure 2:**
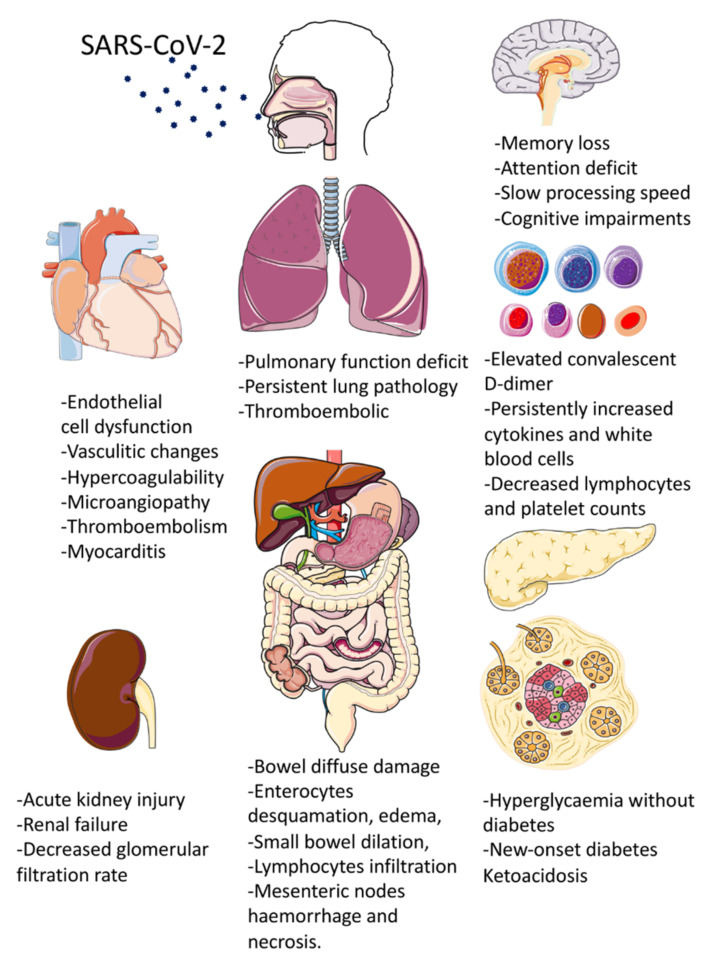
Summary of multi-system clinical presentations of Long COVID-19 Syndrome. (Parts of the figure were drawn and adapted by using pictures from Servier Medical Art (http://smart.servier.com/) (accessed on 20 June 2021), licensed under a Creative Commons Attribution 3.0 Unported License (https://creativecommons.org/licenses/by/3.0/) (accessed on 20 June 2021).

At the time of writing, limited research has been published on this topic. Reports of survivors of SARS-CoV-1 and MERS-CoV infection showed persistent symptoms or underlying organ injury despite “recovery” [15,16,17]. This happens similarly in COVID-19 patients. A recent study showed that the increased risk of Long COVID-19 Syndrome was independent of age and existence of pre-existing medical conditions [18]. This review aims to look into the long-term effects of COVID-19 infection on respiratory, cardiovascular, neurological, and metabolic system of recovered COVID-19 patients (Table 1 and Figure 2), and to make recommendations on the management of Long COVID-19 patients.

### 2.1. Respiratory System

Among patients hospitalized with COVID-19, nearly one-third of the patients met the criteria for acute respiratory distress syndrome (ARDS) [60]. Pathological mechanism has been shown to be the binding between ACE2 and viral structural protein spike S1 domain [61]. Extensive bilateral diffuse alveolar damage with cellular fibromyxoid exudates, desquamation of pneumocytes, and hyaline membrane formations are observed in autopsy reports [19]. Early studies of discharged survivors showed that the most common abnormality was diffusion impairment (51 of 110 cases, 47.2%) [20,21], with another study showing that the majority of patients (54%) had mixed restrictive and low diffusion patterns in pulmonary function tests [62].

At 6 months after acute infection, COVID-19 survivors showed significant pulmonary function deficit characterized by around a quarter reduction of 6-min walking distances compared with the lower range of normal. In the more severely ill groups, more than half of the patients showed irreversible extensive diffuse impairment as verified by computer-tomography scanning [22]. Another study showed a significant portion of patients still had abnormal findings on chest radiography, with another 10% having deteriorating lung pathology radiologically [63]. This was consistent with a European study which showed that persistent symptoms were observed in 41% survivors, while persistent lung pathology was observed in 63% survivors 100 days after COVID-19 onset, presenting as bilateral ground-glass opacifications in the lower lobes [23]. Pulmonary fibrosis with more than 5% affected lung parenchyma were found in 21% of patients [21]. Further analysis showed the numbers of days in intensive care units were related to the extent of lesion on high resolution computer tomography (HRCT), and intubation was associated with signs of fibrosis at follow up (*p* < 0.05). A study showed that hospitalized COVID-19 patients without the need of mechanical ventilations were unlikely to develop long-term pulmonary impairments, thromboembolic complication, or cardiac impairments after discharge [64]. However, this study included only 33 participants. Another study of 8983 patients not requiring hospitalization found that the most important sequels of COVID-19 were dyspnea and venous thromboembolism [65].

SARS-CoV-2 infection poses high risks of thromboembolic events [66,67]. The incidence of pulmonary embolism was high, with 14.2% (95% CI, 7.5–20.8) at the time of hospital admissions for COVID-19, 20.6 to 27% in Intensive Care Unit (ICU) admissions, and 8.3% at conventional ward admissions [67]. Despite recovery from COVID-19 infection, survivors may still live with in-situ thrombosis due to microvascular injury syndrome [68]. Cases of persistent breathless for 5 to 6 weeks due to microvascular injury syndrome after COVID-19 recovery were reported [69]. The pathology was not revealed in ventilation-perfusion mismatches, but picked up only by computer tomography pulmonary angiogram (CTPA) due to unresolved distal small vessel clot. The diagnosis of pulmonary embolism has been difficult due to the overlapping features between pulmonary embolism and severe COVID-19 disease, including dyspnea, high concentration of D-dimers, right ventricular dysfunction or enlargement, and acute respiratory distress syndrome [70]. Contrast dual-energy computer tomography (DECT) is recommended to be a viable means to detect clot and perfusion mismatch since it can identify both venous thromboembolism and small vessel angiopathy [69,71]. High-performance low-field magnetic resonance imaging (MRI) is another valuable tool for repeated evaluation of post-COVID-19 pulmonary damage in some selected cases since it adds no radiation exposure [72].

Pulmonary fibrosis is closely associated with Long COVID-19 Syndrome [24], which can be confirmed radiologically and histologically according to recent studies [25,26,27]. Their radiological findings can be further classified into parenchymal bands, irregular interfaces, reticular opacities and traction bronchiectasis with or without honeycombing [28]. Persistent decline of pulmonary function was observed in these patients despite viral clearance (as shown by PCR testing), and maximal mechanical ventilations by extracorporeal membrane oxygenation (ECMO) [29]. In terms of frequency, a study of 81 survivors showed more than half of them had radiological evidence of pulmonary fibrosis at follow up after discharge [28]. Another study showed 47% of survivors had impaired gas transfer, and 25% of survivors had reduced total lung capacity [20]. Autopsy study was consistent with this conclusion: histological progression of diffuse alveolar damage of fibrosis pattern in 43% of samples [30].

A nationwide study, “UK Interstitial Lung Disease Long COVID19 (UKILD-Long COVID) study”, was launched in the UK in April 2021 to investigate the pathophysiology, clinical progression and treatment strategies [73]. Research data on the optimum rehabilitations of Long COVID-19 Syndrome is lacking.

### 2.2. Cardiovascular System

COVID-19 causes extensive microcirculation disturbance, such as endothelitis, micro-thrombosis, capillary damage, and damage to pericytes, which are vital to capillary integrity and barrier function [31,32,33]. The underlying vascular damage is shown to be associated with cytokine storm and macrophage activating syndrome, causing endothelial cells dysfunction [34,74].

A study identified vasculitis changes in COVID-19 survivors with persistent symptoms: the target-to-blood pool ratio was significantly higher in large vessels, suggesting that the post-COVID-19 vascular inflammation may be responsible for the unexplained symptoms [75]. The micro-vessels were also affected. The impaired oxygen diffusion in lung and tissues may be related to alveolar capillary occlusions [32] and capillary shunting due to changes in alveolar angio-architectures [76], causing hypoxemia and tissue hypoxia.

COVID-19 can also cause hypercoagulability, microangiopathy, thromboembolism, and myocarditis [35,77,78,79]. Cardiac magnetic resonance imaging performed between 11 to 53 days after hospital discharge showed ongoing myocardial inflammation in 60% COVID-19 survivors in Germany; and the myocarditis was independent of chronic comorbidities, duration, and severity of COVID-19 illness [78]. Another study showed that the prevalence of pericarditis or myocarditis 10 weeks after infection was 40%, with 11% of survivors having both pathologies [80]. The major mechanism for myocarditis has been associated with immunological damage; thus, the use of medications to arrest cytokine storm would accelerate the recovery of cardiac function [81]. COVID-19 myocarditis has good prognosis: the majority (81%) of the COVID-19 myocarditis patients survive the acute episode [82]. Since ongoing subclinical myocarditis may evolve into myocardial dysfunction and sudden cardiac death [35], screening of structural and rhythmic abnormality should be done. Common rhythmic abnormality documented in Long COVID-19 patients include atrial fibrillation, supraventricular tachycardia, complete heart block, and ventricular tachycardia [83].

The post-discharge mortality rate of COVID-19 survivors with cardiovascular co-morbidities remain high. In a multicenter study including over 4000 patients in Poland, an overall mortality rate of COVID-19 patients with cardiovascular co-morbidities was 41.9% [84]. The median time to death after discharge was 14 (range 7–30) days. At 2 months after discharge, an additional 5% of the whole study cohort died. Patients with high cardiovascular risks should be referred for clinical reviews and reassessments under specialist care within 1–2 months of discharge [85,86].

### 2.3. Haematological System

A study showed that over 30% survivors had elevated d-dimer and 9.5% had elevated C-reactive protein levels with a median follow-up of 54 days after discharge [63]. Elevated convalescent D-dimer were more common with hospitalized COVID-19 survivors whose age was over 50 years (*p* < 0.001) [36]. The C-reactive proteins returned to normal in more than 90% of survivors at a median of 80.5 days (range 44–155) after initial diagnosis [36].

Cytokines are also shown to be persistently increased 3 months after initial infection [87]. Interleukin-4 (IL-4) and IL-6 are increased in all survivors. In a systematic review and meta-analysis involving 21 studies, increased white blood cells count, decreased lymphocytes and platelet counts, elevated biomarkers of inflammations (IL-6, IL-10), evidence of cardiac muscle injury (cardiac troponin level), liver and renal functions, coagulation profiles, and serum ferritin were strongly associated with progression of COVID-19 [38]. Research data on the trend of these biomarkers in COVID-19 survivors is lacking. More research is required to select the most prognostic biomarkers for monitoring clinical progression of Long COVID-19 patients.

Since COVID-19 may induce endothelitis and systemic inflammations after recovery [66,88], anticoagulants in high-risk patients should be considered. Cardiovascular risk factors should be controlled with the usual guidelines. Venous thromboembolism risk assessment is recommended and the use of thromboprophylaxis with rivaroxaban, betrixaban, or low-molecular weight heparin for high-risk patients are acceptable [77]. Close monitoring of D-dimer levels with IMPROVE D-dimer score is recommended as a part of thromboembolism risk. Age-adjusted D-dimer cut-off level should be further explored to rule out venous thromboembolism in both patients with active COVID-19 infection and survivors [37].

### 2.4. Renal System

Renal impairments have been commonly observed in COVID-19 patients due to the high abundance of ACE2 expression in the kidneys [89]. A systematic review and meta-analysis showed that acute kidney injury (AKI) was observed in half of the non-survivors and less than 1% in survivors [39]. This is consistent with another study which showed that 1.4% of patients experienced renal failure in longer term follow-up of Long COVID-19 Syndrome [90]. Previous studies showed that both chronic and acute kidney injury were associated with significant risks of mortality and morbidity [40,91]. COVID-19 associated estimated glomerular filtration rate (eGFR) declines more rapidly than those without COVID-19 infections [92].

Among discharged survivors with AKI previously, one in three would still depend on renal replacement therapy (RRT) at discharge, and one in six remains RRT dependent 60 days after hospital admission [93]. Longer follow-up duration shows more promising results for restoration of renal functions in survivors with previous AKI: over 90% achieve variable degrees of renal recovery, with over 60% achieving complete recovery [94]. In the management of COVID-19 associated kidney injury, early recognition of kidney involvement and the use of preventive and therapeutic measures to limit AKI and subsequent progression to more severe stages are crucial to reduce morbidity and mortality [95].

Several hypotheses have been postulated to explain the pathophysiology of COVID-19-induced AKI. These include sepsis, renal infarction, respiratory failure-induced AKI, and direct viral invasion of host cells [96]. Around 60% patients experienced sepsis, and 20% experienced septic shock, in the earliest study in Wuhan, China [97]. Kidney infarctions were observed in another study [41]. There is close association between respiratory failure and AKI, showing that nearly 90% patients receiving mechanical ventilations developed AKI [42,98].

### 2.5. Digestive System

The incidence of post-COVID-19 related gastrointestinal symptoms is between 3% and 79%, in various reports [99,100]. The gastrointestinal system is rich in ACE2 and furin expression, a serine protease which cleaves the S-spike protein into S1 and S2, leading to easier attachment of the virion to the ACE receptors and the cell membrane [43,101]. Viral shedding is observed in fecal samples at least 5 weeks after symptoms onset, supporting the viral proliferation and fecal-oral transmission hypothesis [44,45,102]. This results in diffuse damage of the bowel, leading to enterocytes desquamation, edema, small bowel dilation, lymphocytes infiltration, and mesenteric nodes hemorrhage and necrosis. High fecal calprotectin levels were found in patients with persistent diarrhea but without the history of prior inflammatory bowel disease, indicating an underlying intestinal inflammatory process [46]. Studies have suggested the possibility of persistent gastrointestinal dysfunction in various ways: plasma cells and lymphocytic infiltrations into lamina propria of intestinal tissues [103], intestinal dysbiosis [104], and high cytokines level were detected in stool samples [47]. The clinical presentation is similar to irritable bowel syndrome, and the symptoms develop after the resolution of acute COVID-19 infection [105].

Mild impairments in various gastrointestinal organs are observed in low-risk young patients with Long COVID-19 Syndrome (*p* < 0.05) [106]. These include the liver (10%), pancreas (17%), and spleen (6%). The detailed mechanism is still under investigation. Multi-organ injury to hepatobiliary systems may be related to drug-induced liver injury, systemic inflammatory reactions, hypoxia-reperfusion hepatic injury, and possible direct viral injury by SARS-CoV-2 [107]. Typical liver injury pattern can be mixed hepatitic and cholestatic in nature: with elevation of aspartate transferase (AST) and alanine transferase (ALT), gamma-glutamyl transferase (GGT), and alkaline phosphatase (ALP) [108,109]. A 2-month follow-up study showed that the liver enzymes (ALT, AST, GGT, and ALP) remained persistently elevated 14 days after discharge, while the liver functions in the majority of survivors normalized 2 months after hospital discharge [109]. However, patients with liver cirrhosis suffer more mortality compared with those without cirrhosis according to a study involved with 745 patients, suggesting COVID-19 has a significant impact on chronic liver condition [110].

Though pancreatic injury has been observed in COVID-19 survivors [111], the rise of serum amylase and lipase may be due to other causes [49]. Hyperamylasemia may be related to salivary gland inflammations [112], chronic alcoholism, and COVID-19 associated diarrhea [48].

### 2.6. Neurological System

Long COVID-19 Syndrome is associated with mood changes, cognitive difficulties, headache, fatigue, dizziness, memory loss, confusion, and attention deficit [50]. Previous studies on coronavirus SARS-CoV-1 and MERS-CoV show survivors may live with neurological symptoms, such as memory loss, attention deficit, and slow processing speed [51]. A significant proportion of COVID-19 survivors complains of memory loss more than 100 days after hospital discharge [52]. Anatomically, the cognitive impairments and memory loss may be associated with ischemic damages to cerebral white matter [53]. The underlying reason for COVID-19-related neurological damage may be related to blood vessel damage, impaired oxygen supply, viral infiltration into the central nervous system, and inflammatory cytokines-mediated cellular damage [31,113]. Hypoxic injury, microbleedings, and neuronal inflammations in different areas of the brain have been observed in various reports [114,115]. One of the notable sites is the brainstem. The brainstem contains numerous distinct nuclei and subparts that regulate various physiological process: respiratory, cardiovascular, gastrointestinal, and neurological. As neurons do not readily regenerate, brainstem dysfunction may be long-lasting, contributing to Long COVID-19 Syndrome [116]. Radiologically, hypometabolism in various brain areas have been observed in post-COVID-19 patients, indicating underlying SARS-COV-2 related neurotropism [117].

Mitochondrial swellings secondary to hypoxic damage are being observed in Long COVID-19 patients [118]. Neurons with high metabolic demand of oxygen, thus, become dysfunctional, leading to impairments of cognitive functions. This has been similarly observed in other pandemics [119]. The hypometabolism in parahippocampal gyrus, thalamus, and some white matter may be a secondary result of hypoxic damage to these areas, leading to memory loss and cognitive dysfunctions [120].

The multifocal neurological damages in COVID-19 patients result from indirect T-cell and microglia damage in the brain, similar to strokes and neuroinflammatory diseases [54]. Cytokines IL-4 and IL-6 are also shown to be persistently elevated in individuals reported with neurological symptoms [87]. Protein markers related to neuronal dysfunction are increased compared with historic control level. These markers include: amyloid beta, neurofilament light, neurogranin, total tau, and p-T181-tau. The increased markers may be accountable for some psychopathologies, such as anxiety and depression, in COVID-19 survivors [121]. Over 40% of survivors without prior psychiatric conditions live with depression within 90 days of recovery from severe COVID-19 associated respiratory failure [122].

The prevalence of psychological distress is high in the initial phase after discharge [123]. A study of 126 survivors of COVID-19 patients showed 31.0%, 22.2%, and 38.1% of them suffered stress, anxiety, and depression, respectively, suggesting the rate of psychological distress is high in early convalescence [123]. Thus, timely evaluations and treatment are required. Severely ill patients who receive complicated procedures, such as intubations or those who experienced severe complications, may be considered as high-risk of post-traumatic stress disorder [55]. Though evidences about the association between long COVID and post-traumatic stress disorder (PTSD) is lacking, assessing PTSD for these survivors is still recommended [55].

### 2.7. Metabolic System

Patients with Long COVID-19 Syndrome may also present with endocrinopathy, such as diabetes mellitus (DM). As SARS-CoV-2 binds to ACE2 receptors, which are also expressed in pancreatic β-islet cells, it is plausible that the virus may disturb glucose metabolism by intruding the cells. Several decades ago, virus-associated DM was reported on enteroviruses, such as coxsackievirus B [124], rotavirus [125], mumps virus [126], and cytomegalovirus [127]. β-islet cells infected with the viruses triggers phagocytosis by macrophages, thereby triggering autoimmune responses because of the exposure of the antigen from damaged islet cells, which leads to type I DM (T1DM). This autoimmunity promotes anti-viral T-cell memory, which reacts with new infections. As a result, antibodies are likely to target the islet cells, hence aggravating the T1DM. There are also some precedents of SARS coronavirus binding to ACE2 receptors and damaging islets cells [128].

Hyperglycemia without DM and new-onset DM are both associated with a poor course of COVID-19 after excluding risk factors, such as obesity and corticosteroid administration [129]. Newly diagnosed DM cases associated with COVID-19 are mostly T1DM. [56,59]. These patients have favorable outcomes when the infections do not cause hypoxemia, but the outcome is poor for patients with severe COVID-19 [57,130].

In spite of the association between T1DM and the COVID-19, there is still insufficient correlation analysis. A report also shows the association of insulin resistance with poor outcome of COVID-19 by using TyG index, suggesting the possibility of new-onset T2DM because of the infection [58]. Owing to the limited number of reported cases, there may be sampling bias causing unexpected findings or conclusions.

## 3. Recommendation of Follow-Up

**Figure 3:**
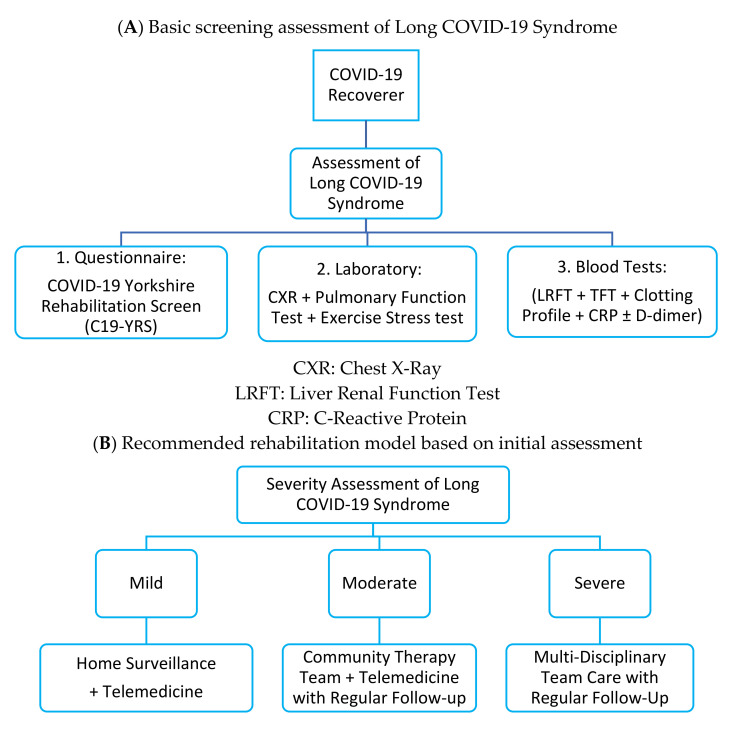
(**A**,**B**) Recommended rehabilitation model for patients with Long COVID-19 Syndrome.

In view of the multi-organ involvement of Long COVID-19 Syndrome, it is recommended that a multi-disciplinary one-stop clinic should be set up to screen, diagnose, and treat affected patients [131] (Figure 3 and Table 2). Specialist expertise and inter-disciplinary collaboration is required for delivery of quality service. Due to the huge number of COVID-19 recovered populations in the foreseeable future, new guidelines to standardize the screening and testing for survivors are needed. These tests may include but are not limited to: screening questionnaires (such as C19-YRS, Yorkshire Rehabilitation Screen), liver and renal function test, thyroid function test, clotting profiles, C-reactive protein, d-dimer, and chest x-ray, and ideally pulmonary function tests and exercise stress test. Previous study showed that a significant proportion of patients have persistently abnormal chest radiographs findings and elevated C-reactive protein and d-dimer levels 2 months after discharge [63].

Proactive identifications of patients with high-risk of Long COVID-19 Syndrome should be advocated. Experience of more than 5 symptoms during the first week of illness is highly associated with Long COVID-19 Syndrome, with an odd ratio of 3.53 (95% CI, 2.76–4.50) [6]. This first-week model is able to differentiate between short-COVID-19 and Long-COVID-19 (total sample size = 2149 patients) with an area under the curve of the receiver operating characteristic curve of 76%, with confirmation from an independent sample of 2472 SARS-CoV-2 patients. Patients who have been more severely ill during their hospital stay have more severe impaired pulmonary diffusion capacities and abnormal chest radiographic imaging manifestations [22]. These patients should be the major target population for long-term rehabilitations.

Several professional bodies have voiced out the need for early detection and rehabilitations of patients with Long COVID-19 Syndrome. The British Thoracic Society recommended follow-up chest radiography for all patients admitted to hospital with COVID-19 infection 3 months after discharge. Those with a history of moderate or severe disease, with clinically persisting symptoms or with radiological abnormalities should be offered further clinical referrals and investigations [134]. Radiological findings of pulmonary fibrosis 4 months after COVID-19 infection are associated with pulmonary function decline and frailty [135]. The Swiss COVID Study Group and Swiss Society for Pulmonology (SSP) formulated a 13-question screening tool to address the diagnosis and are treatment of pulmonary lung damage in Long COVID-19 Syndrome patients [136]. They recommended pulmonary assessment, such as pulmonary function tests, plethysmography, diffusion capacity measurements, and blood gas analysis, in discharged patients with persistent respiratory symptoms after previously PCR-confirmed COVID-19 infections. The SSP strongly recommended rehabilitations in these patients.

Early rehabilitation has been shown to be vital for the improvement of long-term recovery and functional independence of patients [137]; thus, rehabilitations should be started as early as possible [138]. However, there have been reports against early rehabilitations because it may cause intolerable acute desaturation in some previously severe COVID-19 survivors with irreversible pulmonary damage [139,140]. Because of the large populations worldwide, rehabilitation should be planned ahead by the government, healthcare practitioners, and patient groups to address the tremendous needs of rehabilitation for the Long COVID-19 Syndrome [8,141]. Government should provide the demographics of patients for rehabilitations, including comorbidities, complications from intensive care units, and information on various organ dysfunctions. The rehabilitation program has to be personalized and focused to address patient’s specific problems [142]. A recent study has shown that a 6-week respiratory rehabilitation program significantly improves pulmonary functions, quality of life, and anxiety in COVID-19 survivors [143]. Revision of rehabilitation guidelines is required for rehabilitation therapists with a goal to optimize function, minimize disability, and facilitate early return to participation in society. Patient groups should be set up to disseminate rehabilitation information to maximize function and quality of life.

A three-tier rehabilitation service model has been recommended to address different needs of patients [144]. The three levels are specialist multidisciplinary team (MDT) service, community therapy teams, and self-management. The MDT provides specialist skill to address a specific core rehabilitation outcome. The community therapy teams can perform regular follow-up for stable patients. A French study showed that over two-thirds of the non-critical COVID-19 patients were still symptomatic 60 days after initial onset [145]. These patients may be managed conservatively by self-surveillance. Use of telemedicine, checking for adherence to medical therapy, prioritizing high risk patients to assign shorten follow-up period, and close risk assessments are important elements for this self-sustaining system [77].

Standardization of reporting system for COVID-19 symptoms and severity should be established. This facilitates clinical communications and evaluation of disease progressions between healthcare professions [146]. A clinical scoring system that includes functional assessment, self-reported symptoms, prognosis of multiorgan involvement, biomarkers, and radiological findings at different times after discharge should be established [131]. Further standardization on the clinical scoring system is required to stratify the risk level of patients and predict morbidity and mortality of the patients.

## 4. Conclusions

COVID-19 causes multi-organ impairments with a significant number of survivors experiencing Long COVID-19 Syndrome. Early identification of targeted populations and early planning of rehabilitations services are vital to their recovery of functional independence and improvement of quality of life. Multidisciplinary collaborations, standardization of reporting systems, treatment guidelines, and rehabilitations guidelines, and a systematic service provider hierarchy are crucial for prompt follow-up and prioritization of high-risk patients.

## Figures and Tables

**Table 1:** Long COVID-19 Syndrome on various systems: an evidence-based summary.

Systems	Main Diagnosis	Features	Possible Mechanisms	Prognosis	References
Respiratory system	Acute respiratory distress syndrome (ARDS)	Extensive bilateral diffuse alveolar damage with cellular fibromyxoid exudates; desquamation of pneumocytes and hyaline membrane formations; diffusion impairment and pulmonary fibrosis.	SARS-CoV-2 spike S1 domain protein binding to ACE2 receptor; Post Acute Respiratory Distress Syndrome fibrosis with diffuse alveolar damage	Pulmonary function deficit 6 months after infection; extensive diffuse impairment; Long-term in-situ thrombosis	[19,20,21,22,23,24,25,26,27,28,29,30]
Cardiovascular system	Endothelitis; Micro-thrombosis, Capillary damage; hypercoagulability; microangiopathy; thromboembolism; myocarditis; atrial fibrillation; supraventricular tachycardia	Increased target-to-blood pool ratio; capillary disturbance; impaired oxygen diffusion.	Cytokine storm and macrophage activating syndrome-caused endothelial dysfunction.	Majority (81%) of the COVID-19 myocarditis patients survived the acute episode; ongoing subclinical myocarditis may evolve into myocardial dysfunction and sudden cardiac death.	[31,32,33,34,35]
Haematological system	Thromboembolism	Elevated convalescent D-dimer and C-reactive protein levels; persistently increased biomarkers of inflammation.	N/A	Prognostic biomarkers for monitoring clinical progression of Long COVID-19 patients need to be investigated	[36,37,38]
Urinary system	Acute kidney injury; renal failure;	Declined glomerular filtration rate (eGFR); kidney infarction	High abundance pf ACE2 expression in kidneys.	Significant risks of mortality and morbidity	[39,40,41,42]
Digestive system	Gastrointestinal impairment and dysfunction; hepatitic and cholestatic liver injury; pancreatic injury	Bowel diffuse damage; Enterocytes desquamation, edema, small bowel dilation, lymphocytes infiltration and mesenteric nodes hemorrhage and necrosis.	Rich in ACE2 and furin expression; fecal-oral transmission; plasma cells and lymphocytic infiltrations into lamina propria of intestinal tissues.	The liver enzymes remained persistently elevated 14 days after discharge, while the liver functions in majority survivors normalized 2 months after hospital discharge	[43,44,45,46,47,48,49]
Neurological system	Mood changes; cognitive difficulties; headache; fatigue; dizziness; memory loss; confusion; and attention deficit.	Hypoxic injury; microbleedings; neuronal inflammations.	Blood vessel damage, impaired oxygen supply, viral infiltration into the central nervous system and inflammatory cytokines-mediated cellular damage; indirect T-cell and microglia damage in the brain, similar to strokes and neuroinflammatory diseases.	Over 40% survivors without prior psychiatric conditions lived with depression within 90 days of recovery from severe COVID-19 associated respiratory failure, while 70% of them did not receive treatment for depression	[50,51,52,53,54,55]
Metabolic system	Hyperglycaemia without diabetic mellitus; new-onset diabetic mellitus; starvation ketoacidosis	High blood glucose level; impaired glucose metabolism	Intruding pancreatic β-islet cells, triggering autoimmune responses because of the exposure of the antigen from damaged islet cells.	Long-term treatment of diabetic mellitus is needed.	[56,57,58,59]

**Table 2:** Useful investigation tools for patients with Long COVID-19 Syndrome.

Systems	Main Diagnosis	Features	Useful Investigation Tools	Abnormalities to Look for	References
Respiratory system	Acute respiratory distress syndrome (ARDS)	Extensive bilateral diffuse alveolar damage with cellular fibromyxoid exudates; desquamation of pneumocytes and hyaline membrane formations; diffusion impairment.	Pulmonary function tests, high resolution CT, histology; pulmonary angiopathy	Restrictive pulmonary function test; Impaired gas transfer; reduced total lung capacity; fibrotic features on imaging; diffuse alveolar damage on histology; pulmonary vasculature	[20,25,27,28,69,71]
Cardiovascular system	Endothelitis; micro-thrombosis, capillary damage; Hypercoagulability; microangiopathy; thromboembolism; myocarditis; atrial fibrillation; supraventricular tachycardia	Increased target-to-blood pool ratio; capillary disturbance; impaired oxygen diffusion.	Electrocardiogram; Echocardiography; coronary angiography and cardiac catheterization; chest X Ray; electron-beam computed tomography; cardiac MRI.	Microcirculation disturbance; Increased target-to-blood pool ratio; impaired oxygen diffusion; myocardial inflammation; rhythmic abnormality	[31,32,33,34,35]
Haematological system	Thromboembolism	Elevated convalescent D-dimer and C-reactive protein levels; Persistently increased biomarkers of inflammation.	Venipuncture for blood tests of D-dimer and C-reactive protein; duplex ultrasound for lower limb clots; CT-pulmonary angiogram for pulmonary embolism; electrocardiogram, echocardiography, coronary angiography and cardiac catheterization for evidence of cardiac or pulmonary embolism	Thrombocytopenia; blood cell abnormalities	[132,133]
Urinary system	Acute kidney injury; renal failure;	Declined glomerular filtration rate (eGFR); kidney infarction	Urine analysis; glomerular filtration rate; ultrasound scanning; MRA; renal biopsy.	Early recognition of kidney involvement; kidney injury; renal infarction	[40,41,96]
Digestive system	Gastrointestinal impairment and dysfunction; Hepatitic and cholestatic liver injury; pancreatic injury	Bowel diffuse damage; Enterocytes desquamation, edema, small bowel dilation, lymphocytes infiltration and mesenteric nodes hemorrhage and necrosis.	Barium beefsteak meal; colorectal transit study; computed tomography scan (CT or CAT scan); defecography; Lower gastrointestinal series; MRI; magnetic resonance cholangiopancreatography (MRCP); oropharyngeal motility (swallowing) study.	Bowel damage; high fecal calprotectin level; gastrointestinal dysfunction; liver injury; pancreatic injury; hyperamylasemia	[43,44,45,46,47,48,49]
Neurological system	Mood changes; cognitive difficulties; headache; fatigue; dizziness; memory loss; confusion; and attention deficit.	Hypoxic injury; microbleedings; neuronal inflammations.	CT scan; electroencephalogram; MRI; electrodiagnostic tests, such as electromyography (EMG) and nerve conduction velocity (NCV); positron emission tomography (PET); arteriogram (angiogram); lumbar puncture; evoked potentials.	Neurological symptoms; ischemic damages to cerebral white matter; blood vessel damage; hypoxic injury, microbleedings, and neuronal inflammations in different areas of the brain; brian hypometabolism	[50,51,52,53,54,55]
Metabolic system	Hyperglycaemia without diabetic mellitus; new-onset diabetic mellitus; starvation ketoacidosis	High blood glucose level; impaired glucose metabolism	Blood tests for blood glucose and HbA1c level; plasma amino acid analysis; plasma Carnitine level; plasma acylcarnitine profile; plasma C-peptide level, Urine organic acid analysis; Urine and plasma ketone analysis	Impaired glucose metabolism; increased ketone body	[56,57,130]

## Data Availability

Data policy is not applicable in this review. All data can be retrieved from electronic databases (PubMed, Google Scholar, Scopus, Embase, Cochrane).
